# Lyophilized Gelatin@non-Woven Scaffold to Promote Spheroids Formation and Enrich Cancer Stem Cell Incidence

**DOI:** 10.3390/nano12050808

**Published:** 2022-02-28

**Authors:** Jingjing Fu, Feng Chen, Huihui Chai, Lixia Gao, Xiaohui Lv, Ling Yu

**Affiliations:** 1 School of Preclinical Medicine, North Sichuan Medical College, Nanchong 637000, China; fujingjing1991@nsmc.edu.cn; 2 Institute for Clean Energy & Advanced Materials, School of Materials & Energy, Southwest University, Chongqing 400715, China; cf19950629@email.swu.edu.cn (F.C.); chh0221@email.swu.edu.cn (H.C.); lvxiaohui@email.swu.edu.cn (X.L.); 3 National & Local Joint Engineering Research Center of Targeted and Innovative Therapeutics, Chongqing Key Laboratory of Kinase Modulators as Innovative Medicine, College of Pharmacy & International Academy of Targeted Therapeutics and Innovation, Chongqing University of Arts and Sciences, Chongqing 402160, China; LixiaGao@cqwu.edu.cn; 4 Guangan Changming Research Institute for Advanced Industrial Technology, Guangan 638500, China

**Keywords:** tumor spheroid, gelatin, non-woven fabric, lyophilized, cancer stem cell

## Abstract

A gelatin@non-woven fabric (gelatin@NWF) hybrid scaffold with tailored micropore structures was fabricated by lyophilizing, using gelatin to support cells and the NWF matrix as a frame to enforce the mechanical stability of gelatin. By freezing the gelatin and NWF hybrid in liquid nitrogen and subsequently lyophilizing and crosslinking the process, the gelatin@NWF scaffold was prepared to support cell growth and promote cell aggregation and spheroids’ formation. The results indicated that by tuning the lyophilizing temperature, the micropore size on the gelatin could be tailored. Consequently, tumor spheroids can be formed on gelatin@NWF scaffolds with honeycomb-like pores around 10 µm. The cell spheroids formed on the tailored gelatin@NWF scaffold were characterized in cancer stem cell (CSC)-associated gene expression, chemotherapy drug sensitivity, and motility. It was found that the expression of the CSC-associated biomarkers SOX2, OCT4, and ALDH1A1 in gene and protein levels in DU 145 cell spheres formed on gelatin@NWF scaffolds were significantly higher than in those cells grown as monolayers. Moreover, cells isolated from spheroids grown on gelatin@NWF scaffold showed higher drug resistance and motility. Tumor spheroids can be formed on a long-term storage scaffold, highlighting the potential of gelatin@NWF as a ready-to-use scaffold for tumor cell sphere generation and culturing.

## 1. Introduction

Two-dimensional (2D) cell culture, a well-established methodology, facilitates the understanding of tumor biology and accelerates drug discovery and development research. Yet, the rapid, uncontrolled growth phenotype of 2D cell culture was challenged by lacking an in vivo-like microenvironment, cell–cell contacting and nutrient distribution, etc. Increasing debates argued that those limitations, in mimicking the physiological and pathological conditions, might partially account for the high discrepancy rate between in vitro and in vivo tests [1,2,3,4,5]. Thus, growing interests and attention have been attracted by three-dimensional (3D) cell culture among clinicians, researchers, and the pharmaceutical industry in the part of its potential in reassembling complexity and heterogeneity of microenvironment to mimic in vivo conditions [6,7]. Compared to 2D tumor cell culture, tumor spheroids are unique in recapitulating tissue architectures, mimicking the nutrition, metabolic, and proliferative gradients of in vivo tumors, and demonstrating clinically relevant chemoresistance [8,9,10,11]. More importantly, studies have revealed that tumor spheroids served as surrogate systems to evaluate cancer stem cell (CSC)-related characteristics in vitro [12,13]. Yamazaki et al. cultured liver cancer and breast cancer cell lines on a polymer thin film to promote tumor spheroid formation. They found that tumor cells of spheroids showed a significant increase in the expression of CSC-associated genes and acquired increased drug resistance compared with 2D monolayer-cultured controls [14]. A similar enrichment of CSC contents has been witnessed from glioma and prostate spheroids [15,16,17]. Considering the significance of CSC in tumor metastasis and recurrence, tumor spheroids are a much better choice than the 2D cell culture model to investigate the therapeutic efficacy in targeting CSC. Hence, tumor spheroids are not only excellent in reflecting the complex structures of solid tumors but are also prominent in evaluating the targeting-CSC efficacies of anti-tumor drugs. 

Methodologically, tumor spheroids are assembled either scaffold-free, such as hanging drop, liquid-over-lay methods, rotating wall vessels, or scaffold-based approaches [18,19,20]. A scaffold with fibrous structures is explored as a candidate to support 3D cell culture due to its porous structures for nutrient diffusion and larger surface area for cell anchoring and proliferation. In scaffold-based approaches, tumor spheroids can be formed on platforms that can mimic the extracellular matrix (ECM) architecture and participate in regulating the biological behavior of tumor cells. The advantages of scaffold-based spheroids culture include (1) high throughput preparation, (2) compatibility with a variety of culture vessels, (3) customized physical and chemical properties, (4) diverse material sources (natural biomaterials and synthetic materials) [19,21,22]. For instance, Marziyeh et al. reported that 3D HT29 spheroids could be formed on Col/PLCL and Gel/PLCL nanofibers with uniform morphology and smooth surface [23]. Xu et al. found that the 3D porous chitosan–alginate scaffolds can support PC-3 spheroids growth by adjusting scaffold stiffness to mimic stages of metastatic progression [24]. Better yet, the scaffold can also provide anchor points for tumor spheroids, which is friendlier for handling and bio-analysis subsequently.

Gelatin, a protein derived from collagen of various animal bodies, has been playing an essential role in cell culture and tissue engineering. Biologically, gelatin may cue cells to orientate and organize themselves into a definite pattern to form in vivo characteristic architectures. Gelatin is generally added to substrate materials to create a relatively stable shape for cell analysis [25,26,27]. In our previous study, we prepared a gelatin@polypropylene non-woven hybrid scaffold in considering of importance of hydrogel to support cells and polypropylene matrix as a frame to enforce the mechanical stability of hydrogel. By freezing the gelatin and polypropylene at −80 °C and subsequently freeze-drying and crosslinking process, a free-standing, ready-to-use gelatin@polypropylene hybrid scaffold was prepared to support cell growth and assemble multi-layer cell cultures [28]. The merits of the free-standing and ready-to-use properties of the hybrid scaffold drive us to think about the potential of modulating the cell behavior on the scaffold by tailoring the micro-structures/architecture of the scaffold, ideally, to promote cell aggregation and spheroid formation. To this end, gelatin concentrations and the lyophilizing conditions were systematically investigated. The relationship between micro/structures and the cell response was analyzed. The cell aggregates formed on the tailored gelatin@NWF scaffold were characterized in CSC-associated gene expression, chemotherapy drug sensitivity, and motility. 

## 2. Experimental Method

### 2.1. Materials and Reagents

Human prostate cancer cell DU145 was obtained from the Cell Bank of Chinese Academy of Sciences, Shanghai. Cells were maintained in DMEM culture medium (Gibco, Grand Island, NY, USA) containing 10% fetal bovine serum (Gibco, Grand Island, NY, USA), penicillin (100 U/mL), and streptomycin (100 g/mL). Polypropylene non-woven fabric (NWF) was purchased from Huaxia Nonwoven Co., Ltd. (Shanghai, China). Gelatin was from Bioengineering Co., Ltd. (Shanghai, China). Moreover, 1-ethyl-(3-two methyl amino propyl) carbon two imide hydrochloride (EDC), N- hydroxysuccinimide (NHS) were purchased from Aladdin Biochemical Technologies Inc. (Shanghai, China). Doxorubicin (DOX), crystal violet staining solution, methylthiazolyldiphenyl-tetrazolium bromide (MTT), and CCK-8 kit were purchased from Beyotime Biotechnology (Beijing, China). Accutase™ stem cell dissociation reagent and fetal bovine serum (FBS) were from Gibco (Grand Island, NY, USA). Prime Script RT Reagent Kit with gDNA Eraser (Perfect Real Time), MiniBEST Universal RNA Extraction Kit, and SYBR^®^Premix Ex Taq™ II (Tli RNaseH Plus) were from TaKaRa (Tokyo, Japan). Rabbit anti- Oct4, anti- ALDH1 A1, anti- Sox2, GAPDH, and HRP-labeled anti-rabbit IgG antibodies were purchased from Cell Signaling Technology (Danvers, MA, USA). All other chemicals were acquired from Sigma-Aldrich (St. Louis, MO, USA) unless otherwise indicated. All solutions were prepared with deionized (DI) water produced by PURELAB flex system, ELGA Corporation.

### 2.2. Gelatin@NWF Hybrid Scaffold Fabricated by Lyophilizing

Preparation of gelatin@NWF scaffolds was started by dropping gelatin solution onto the polypropylene NWF specimen. First, gelatin was dissolved in DI water at 60 °C under 1000 rmp/min agitation. The homogenous gelatin solution was cast onto the NWF specimen. The gelatin-covered NWF specimen was immediately put into liquid nitrogen for 30 min. Then, the samples were freeze-dried at −80 °C for 24 h. After that, the lyophilized gelatin@NWF was immersed in EDC/NHS (50 mM/100 mM) crosslinking agent for 12 h. Finally, the gelatin@NWF hybrid scaffold was dried in an oven at 60 °C and stored at 4 °C for later use.

As a control group, the gelatin-covered NWF specimen was frozen in a −80 °C freezer for 6 h, and then freeze-dried at −80 °C for 24 h. After the subsequent crosslinking treatment detailed above, the obtained scaffold was named gelatin@NWF (−80 °C).

### 2.3. Characterization of the Lyophilized Gelatin@NWF Scaffold 

#### 2.3.1. Morphology

The surface morphology of the gelatin@NWF scaffold was characterized by field emission scanning electron microscopy (FESEM, JSM-7800F; JEOL Tokyo, Japan). The samples were coated with platinum for 1 min before measurement. The scaffolds were also examined under a microscope (TS100-F; Nikon, Tokyo, Japan).

#### 2.3.2. Porosity

The porosity of the gelatin@NWF scaffold was measured by the liquid displacement method [29]. DI water was used as the displacement liquid. The porosity (P) of the gelatin@NWF scaffold was obtained by:

(1) $$P\%=\left(V_{1}-V_{3}\right)/\left(V_{2}-V_{3}\right)\times 100$$

where *V*_1_ is the original volume of the water, *V*_2_ is the water volume after the scaffold immersion, and *V*_3_ is the remaining water volume after removing the scaffold. 

#### 2.3.3. Swelling

The swelling index was measured according to the literature [30]. The scaffold at a diameter of 0.6 cm was weighed and then immersed in water. Every 10 min, the samples were carefully picked up and weighed to determine the amount of water soaked in. The swelling percentage of the scaffold was calculated as follows:

(2) $$\mathrm{Swelling}\%=\left(W_{2}-W_{1}\right)/W_{1}\times 100$$

where *W*_1_ and *W*_2_ are the weight of the scaffold before soaking and after removing out from the water, respectively. The experiments were repeated six times under the same conditions.

#### 2.3.4. Mechanical Strength

The mechanical strength test evaluates the material by tensile strength (TS) and elongation at break (EAB). This study used a universal testing machine (model WDW-211, Zhongzheng Testing Machine Manufacturing Co.Ltd, Jinan, China) to test the TS and EAB of gelatin@NWF scaffold and gelatin film. The specimens with a size of 2 cm × 9 cm were clamped with the initial grip length of 7 cm and pulled at the extension rate of 500 mm/s. TS and EBA are calculated as follows:

TS = F/S EAB % = (L_1_ − L_0_)/L_0_ × 100 (3)

where F is the maximum load (N) before sample breaking, S is the cross-section area (m^2^) of the sample, L_0_ (cm) is the initial grip length (1 cm), and L1 is the maximum grip length before sample breaking. The values were expressed as the means ± standard error (*n* = 7).

### 2.4. Cell Compatibility of Gelatin@NWF Scaffold

#### 2.4.1. MTT Cell Growth Assay

Prostate cancer cell DU 145 was used as a model cell to evaluate the as-prepared scaffold’s cell computability. First, the MTT method was conducted to measure the cell growth on the gelatin@NWF scaffold. In brief, 100 µL cells (5 × 10^5^/mL) were seeded on the gelatin@NWF scaffold (diameter = 0.6 cm). After incubation in a CO_2_ incubator for 1 day, 3 days, and 5 days, MTT solution (0.5 mg/mL) was added to each substrate and incubated for 4 h. Finally, dimethyl sulfoxide (DMSO) was added to dissolve the living cell catalyzed purple precipitate. The absorbance at 570 nm was measured in Gene^®^ ELx800TM microplate reader (Hong Kong, China) with a reference wavelength of 630 nm. All experiments were performed three independent times.

#### 2.4.2. Crystal Violet Staining

A total of 100 µL cells (5 × 10^5^/mL) were seeded on the gelatin@NWF scaffolds (d = 0.6 cm). After 24 h of incubation, the non-adhesive cells were washed off with 0.01 M pH 7.4 PBS solution. The adherent cells were fixed in 4% paraformaldehyde and stained with crystal violet (5 ng/mL). Finally, the adherent cells of six randomly selected fields per scaffold were imaged using TS100-F microscope. All experiments were performed three times in triplicate.

### 2.5. Biomarker Levels of Cells Grown on Gelatin@NWF Scaffold

The expression of ALDH1A1, OCT 4, and SOX2 in the cells grown on gelatin@NWF scaffold was studied in gene and protein level by real-time fluorescent quantitative PCR (RT-qPCR) and Western blot, respectively. In brief, 100 µL cells (5 × 10^5^/mL) were seeded on the gelatin@NWF scaffold (diameter = 0.6 cm). After 24 h incubation, the scaffold with DU 145 was washed with 0.01 M PBS solution and transferred to a new cell culture plate keeping culture for 5 and 7 days. Then the adherent cells were collected by Accutase™ stem cell dissociation reagent as described in the literature [31,32]. RT-qPCR and Western blot analyzed the cells as follows. The cells growing in a standard cell flask were tested simultaneously as a 2D cell control.

#### 2.5.1. Quantitative Real-Time PCR

The total RNA was isolated with the MiniBEST Universal RNA Extraction Kit according to the protocol. The RT-qPCR condition is detailed in the previous study [33]. PCR primers for ALDH1 A1, OCT4 and SOX2 genes were designed and validated per the guidelines recommended by the MIQE (Supplementary Materials Table S2) [34]. Glyceraldehyde-3-phosphate dehydrogenase (GAPDH) was used as an internal control. The relative expression levels of each gene in cells were normalized in comparison with the GAPDH gene using the 2^−ΔΔCt^ method. Three independent experiments were performed.

#### 2.5.2. Western Blot

Cells collected from gelatin@NWF were lysed by 0.1% Triton X-100 in PBS containing 2% β-mercaptoethanol. The cell lysates were resolved SDS-PAGE and transferred onto the PVDF membrane. The Western blot condition is detailed in the previous study [35]. The primary antibodies used were monoclonal anti-GAPDH antibody (mAb) (1:1000), Oct4 (1:1000), ALDH1a1 (1:1000), and Sox2 (1:1000). The band density was analyzed using ImageJ V1.8.0.112(NIH, Bethesda, MD, USA). The relative expression levels of each protein in cells were normalized to GAPDH expression.

### 2.6. Migrating Capability of Cells Grown on Gelatin@NWF Scaffold

Boyden chamber assay was conducted to analyze the DU 145 cells grown on gelatin@NWF scaffold. First, 100 µL cells (5 × 10^5^/mL) were seeded on the gelatin@NWF scaffold and cultured for 5 days. Then the cells grown on gelatin@NWF scaffold were retrieved by using Accutase™ stem cell dissociation reagent. Next, the harvested live cells were analyzed by Boyden chamber assay. In brief, 600 μL complete culture medium containing 20% FBS was added into the lower chamber of the transwell plate. Subsequently, 100 µL single-cell suspension (5 × 10^4^ cells/mL) was added into the transwell chamber and culture at 5% CO_2_ and 37 °C for 48 h. Then, the transwell membranes were stained by crystal violet. Six randomly selected fields per transwell were imaged using a microscope to quantify the migrating cells. The experiments were repeated three times independently. The cells growing in a standard cell flask were tested simultaneously as a 2D cell control.

### 2.7. The Effect of Chemotherapy Drug DOX on Cells Grown on Gelatin@NWF Scaffold

Doxorubicin (DOX) was used as a model drug. A series concentration of DOX, as indicated, was prepared to treat the cells. Drug sensitivity tests were carried out on DU 145 cells cultured on gelatin@NWF scaffold for 7 days. The cells growing in a standard tissue culture plate were tested at the same time as a 2D cell control. First, the cell spheroids collected from gelatin@NWF were digested by using Accutase™ stem cell dissociation reagent. Then, the single-cell suspension (100 μL, 1 × 10^4^/mL) was cultured in 96-well microplate for 24 h. Next, the culture medium was replaced with 100 μL different concentrations of DOX (0, 0.25, 0.50, 0.75 and 1.00 μM) and incubated for another 48 h. Finally, the alive cells were quantified by CCK-8 assay. In short, a fresh culture medium containing CCK-8 reagent was added to each well and incubated at 37 °C for 4 h. The absorbance of each well was measured at 490 nm with a microplate reader. All experiments were repeated three times in triplicates.

### 2.8. Statistical Analysis

All experiments were performed three times in triplicate. Data are expressed as the mean ± standard deviation. Results were analyzed with the one-way ANOVA with a post hoc test using SPSS (International Business Machines Corporation, Armonk, NY, USA) and Origin Statistic software (OriginLab Corporation, Northampton, MA, USA). P values less than 0.05 were considered statistically significant.

## 3. Result and Discussion

### 3.1. Tailored Honeycomb-like Micro-Pores Formed by Lyophilizing Gelatin@NWF in Liquid Nitrogen

**Figure 1:**
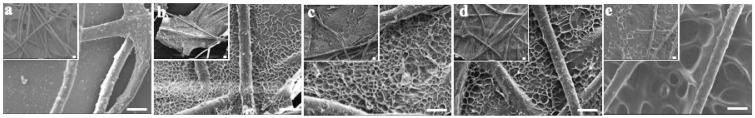
FESEM characterization of gelatin@NWF scaffold prepared at different conditions. (**a**) NWF, (**b**) 6% gelatin@NWF, (**c**) 9% gelatin@NWF, (**d**) 12% gelatin@NWF, (**e**) 9% gelatin@NWF (−80 °C), scale bar = 50 μm.

First, the impact of the lyophilizing condition on gelatin@NWF scaffold’s microstructures was investigated. Homogenous gelatin solution was cast on NWF fibrous and frozen in liquid nitrogen or −80 °C. FESEM examined the morphology of the lyophilizing prepared scaffolds. As the FESEM results shown in Figure 1a, gaps can be clearly observed from the original NWF scaffold. Only part of the gaps between NWF fibers was filled when the 6% gelatin was applied (Figure 1b). When the gelatin concentration was 9% and 12%, gelatin film with honeycomb-like pores could be observed between NWF fibers (Figure 1c,d). By contrast, for the scaffold prepared by freeze-drying at −80 °C, relatively larger pores could be observed. The average size of the honeycomb-like pores on gelatin film was quantified based on the FESEM images. When the gelatin concentration increased from 9% to 12%, the pore size did not change significantly (9% vs. 12%: 10.60 ± 1.14 µm vs. 11.91 ± 1.78 µm, *p* > 0.05). While the pore size was larger than 28.05 ± 3.91 µm when the scaffold was frozen at −80 °C (Figure 1e), the number is in line with a previous study [28]. We speculated that the gelatin pore size is determined by the ice crystal’s nucleation rate and the speed of crystal growth. When the freezing temperature is lower (liquid nitrogen), the nucleation rate of ice crystals is faster than its growth rate, so a large number of smaller ice crystals are formed. Subsequently, after the sublimating stage, smaller honeycomb-like pores formed on the gelatin film. When the freezing is at -80°, the ice crystal growth rate is more significant than its nucleation rate, resulting in a large crystal formation. After the freeze-drying process, relatively larger pores were left on the gelatin film.

**Figure 2:**
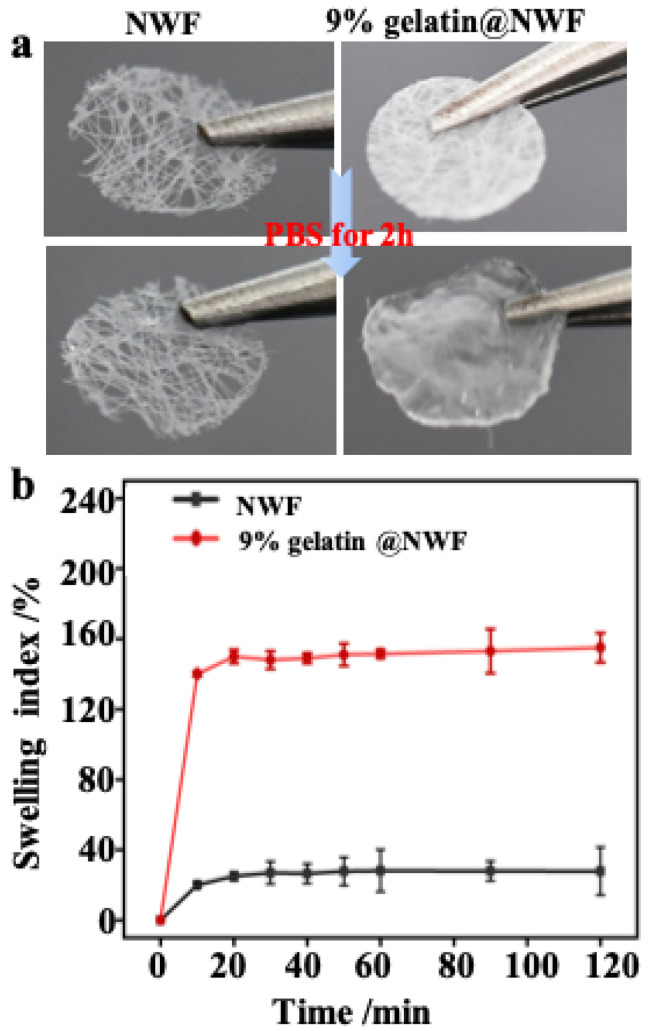
Swelling property of gelatin@NWF scaffold: (**a**) photographs of the scaffold before and after swelling; (**b**) swelling kinetics curve.

The free-standing and ready-to-use potential of the liquid nitrogen-prepared gelatin@NWF scaffolds were investigated. As shown in Figure 2a and Supplementary Materials Figure S1a, the non-woven substrate used in this work is a thin layer of fabric with a thickness of ~36 μm. The void ratio of the pristine NWF is about 88%. The freeze-drying process-fabricated gelatin@NWF is a white sheet, which can be easily handled (Supplementary Materials Figure S1b). After immersion in 0.01 M PBS (pH 7.4) for 2 h, the gelatin@NWF scaffold changes from white to transparent, with the thickness increased by 8–10 times (Figure 2a). The swelling kinetics show that the maximum swelling rate of the gelatin@NWF scaffold was achieved at 10 min. The swelling rate does not change significantly in the following time, suggesting the scaffold can adsorb cell culture medium quickly to support the cell growth (Figure 2b). Next, the mechanical properties of the composite were evaluated by tensile strength and elongation at break (Supplementary Materials Table S1). The tensile strength and elongation at the gelatin break are 0.81 ± 0.14 MPa and 32.12 ± 4.51%, respectively. While for the lyophilized gelatin@NWF scaffold, the tensile strength increases to 0.98–1.23 MPa, and the elongation at break increases from 32.12% to 46.77%. The results are in line with the previous study in which the scaffold was prepared by freeze-drying at −80 °C [28]. Compared with pure gelatin scaffold, blending with NWF can improve the overall mechanical strength, benefiting the long-term culture and assembling a multi-layer gelatin@NWF platform for 3D cell culture.

### 3.2. Tailored Honeycomb-like Micro-Pores Modulate Cell Aggregating on Gelatin@NWF Scaffolds

**Figure 3:**
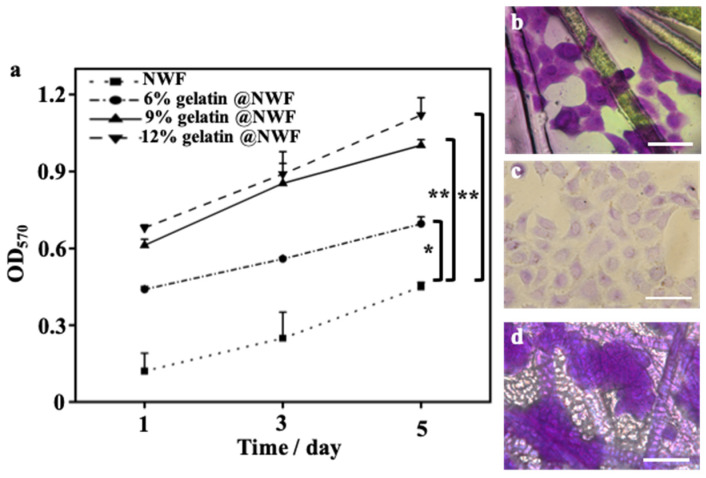
Cell compatibility of gelatin@NWF. (**a**) MTT assay characterized DU 145 cells proliferation on hybrid scaffold prepared from different concentrations of gelatin, * denotes *p* < 0.05, ** denotes *p* < 0.01; bright-field images of crystal violet-stained DU 145 cells grown on (**b**) gelatin@NWF (−80 °C), (**c**) tissue culture plate (TCP), and (**d**) gelatin@NWF, scale bar = 50 µm.

**Scheme 1:**
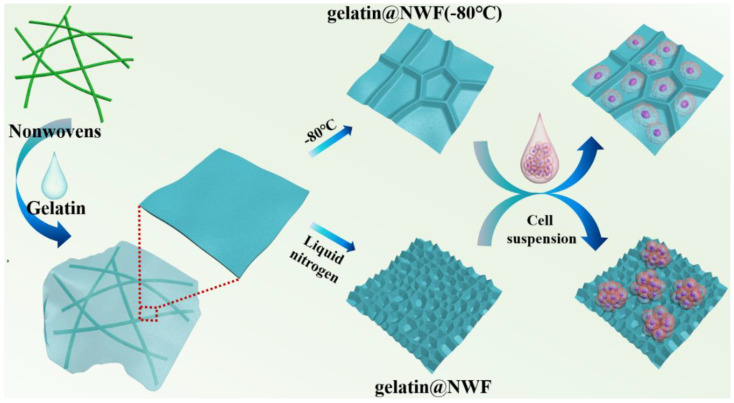
Illustration of the preparation of gelatin@NWF scaffold for tumor spheroid formation.

Primary prostate cancer is the second most common malignant tumor worldwide and the sixth leading cause of cancer death among men. The incidence rate of prostate cancer is still rising yearly [36]. The prostate cancer spheroid culture has been suggested as a useful technique in prostate cancer research [37,38]. DU 145 cells were derived from prostate cancer brain metastasis and have been wildly used for pharmacological and metabolic studies [39,40]. DU 145 cells were used as a cell model in the following experiment. Du 145 cells growing on gelatin@NWF scaffolds were quantified by MTT assay. As shown in Figure 3a, liquid nitrogen prepared gelatin@NWF scaffolds support more cell growth than pure NWF scaffolds. The higher absorbance value in MTT assay is given by hybrid scaffolds prepared from 9% gelatin and above. This might be due to the gelatin filling covering the gaps between NWF fibrous matrix, providing more surface for cell anchoring and growing. Next, cell morphology on the lyophilizing prepared gelatin@NWF scaffold was studied. As shown in Figure 3b, cells attach and spread on gelatin@NWF (−80 °C) scaffold prepared by freezing at −80 °C. The morphology of DU 145 cells is very similar to the cells grown on a tissue culture plate (Figure 3c). However, spreading cells are not noticeable on the liquid nitrogen-freezing gelatin@NWF scaffold. Instead, cells turned to aggregate. Moreover, cell aggregates are mainly distributed on the gelatin fillings between NWF fibers (Figure 3d and Figure S2), suggesting the physical architecture or morphology of gelatin@NWF scaffold plays an essential role in regulating and controlling cell behavior. As shown in Figure 1, the pores on gelatin@NWF are in the range of 10.60–15.47 μm. In comparison, the average pore size on gelatin@NWF (−80 °C) is 28.05 ± 3.91 µm. We speculated that when the pore size is larger than the cell size, the cell will recognize it as a plane and spread growth. On the contrary, the limited space will restrain the cell spreading; thus, cells turn to form cell aggregation and even spheres (Scheme 1). 

**Figure 4:**
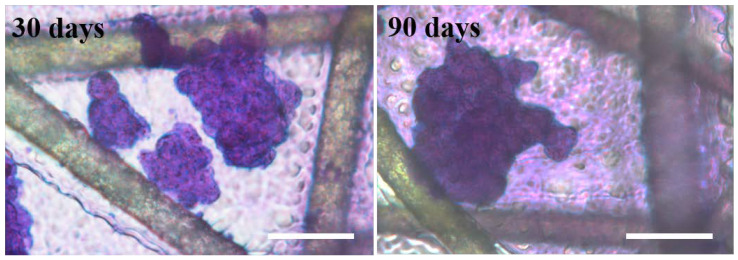
Stability of gelatin@NWF scaffold. DU 145 cells were cultured on the scaffolds, which were stored for 30 and 90 days, respectively, scale bar = 50 μm.

Moreover, the scaffold was stored at 4 °C for 30 and 90 days and then used as a substrate for cell culture. The cell behavior on those long-term storage scaffolds was studied. As shown in Figure 4, DU145 cells still form tumor spheres on the long-term storage gelatin@NWF scaffold, which further indicates the lyophilized scaffold has good functional stability, highlighting its potential as a ready-to-use scaffold for tumor cell sphere generation and culturing.

### 3.3. Elevated Cancer Stem Cell-Related Biomarker in Tumor Spheres Grown on Gelatin@NWF Scaffold

**Figure 5:**
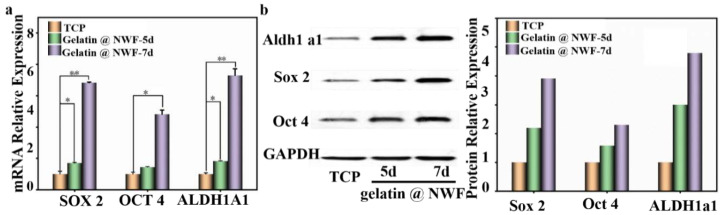
An elevated level of putative CSC markers in cells grown on gelatin@NWF scaffold. (**a**) RT-qPCR characterized relative mRNA expression of OCT4, SOX2, and ALDH1A1 in cells grown on gelatin@NWF scaffold for 5 and 7 days, * denotes *p* < 0.05, ** denotes *p* < 0.01; (**b**) Western blot analysis to compare the Sox2 (35 kDa), Oct4 (45 kDa), and ALDH1A1 (55 kDa) level compared to GAPDH (36 kDa) in cells grown on TCP and gelatin@NWF scaffold for 5 and 7 days.

The biological characters of the cell aggregates formed on tailored gelatin@NWF scaffold were investigated by analyzing cancer stem cell-related biomarkers’ gene and protein expression level, chemo-sensitivity, and migration ability. Gelatin@NWF scaffold prepared from 9% gelatin was applied in the following cell assays. Biomarkers such as OCT 4 and SOX 2 play an essential role in regulating tumorigenesis and renewal, thus being associated with cancer stem cells [35,41]. First, the gene expression levels of those CSCs related biomarkers was quantified by RT-qPCR. As shown in Figure 5a, compared with the cells cultured in the tissue culture plate, the self-renewal and pluripotency related genes OCT4 and SOX2 in DU145 cells cultured on gelatin @NWF scaffold for 7 days show a significant upward trend with an increase of 4.82 ± 0.21 and 6.83 ± 0.04 folds, respectively. Aldehyde dehydrogenase 1A1 (ALDH1A1) is a critical enzyme that synthesizes retinoic acid. Increasing evidence supports that it is a crucial CSC marker and a valuable predictor of the progression of solid tumors such as breast cancer, prostate cancer, and colorectal cancer [42]. From the RT-qPCR results, the expression of ALDH1 A1 also increased rapidly in the spheres grown on gelatin@NWF scaffold after 7 days (gelatin@NWF-7 d vs. TCP: 7.31 ± 0.42 µm folds, *p* < 0.01). Next, by using Western blot analysis, the protein expression level of CSC-related markers was identified. As shown in Figure 5b, protein expression of Oct 4, Sox 2, and ALDH1a1 significantly increased to 2.31 folds, 3.90 folds, and 4.80 folds in cells grown on gelatin@NWF scaffold for 7 days compared to 2D cultured DU 145 cells. The RT-qPCR and Western blot assay proved that cells grown on gelatin@NWF scaffold have a spheroid morphology and an increased CSC-related marker expression, which strongly indicated that gelatin@NWF scaffold has great potential to become a platform for cancer stem cell enrichment in vitro.

### 3.4. Increased DOX Resistance and Cell Motility of Cells Grown on Gelatin@NWF Scaffold

**Figure 6:**
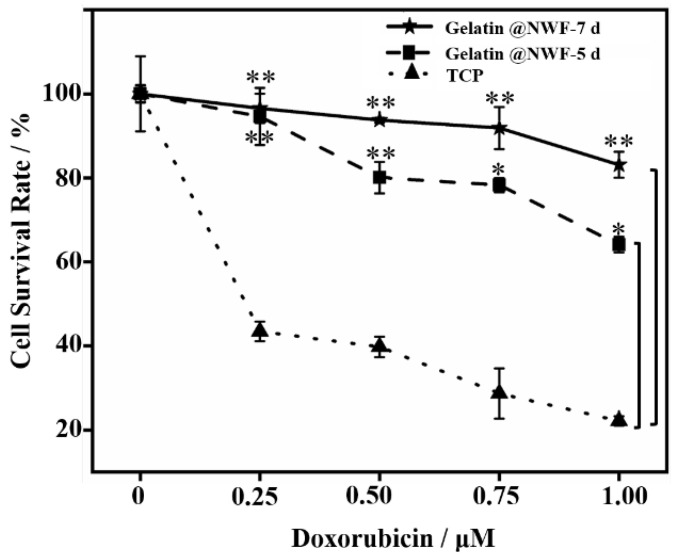
Increased drug resistance of cells grown on gelatin@NWF scaffold. DU 145 cells were cultured on gelatin@NWF for 5 and 7days. Then cells were collected and treated by doxorubicin (0.25∼1 μM) for 48 h. The CCK-8 proliferation assay was conducted to measure cell growth. * denotes *p* < 0.05, ** denotes *p* < 0.01.

The resistance of cancer stem cells to chemotherapeutic drugs is a hallmark inducement for the poor effect of chemotherapy [43]. In this study, a broad-spectrum chemotherapy drug Doxorubicin (DOX), was used as a model drug to evaluate the drug sensitivity of tumor cells grown on gelatin@NWF scaffold. CCK-8 cell proliferation results show that the survival rate of DU 145 cells cultured on gelatin@NWF fabric is significantly higher than cells grown in a tissue culture plate (*p* < 0.05). The results suggest cells grown as a sphere on gelatin@NWF scaffold have a more substantial tolerance to chemotherapy drugs (Figure 6). In addition, when increasing the time of cell growing on gelatin@NWF scaffold to 7 days, the drug sensitivity of the cells on gelatin@non-woven decreased sharply. Specifically, when DOX concentration was 1 μM, the survival rate of cells cultured on gelatin@non-woven for 7 days was 83.12%. The survival rate of cells cultured on gelatin@non-woven for 5 days was around 68%, while the survival rate of cells cultured on TCP was only 12.2%. This result may be due to an increase in the proportion of cancer stem cells in tumor spheres.

**Figure 7:**
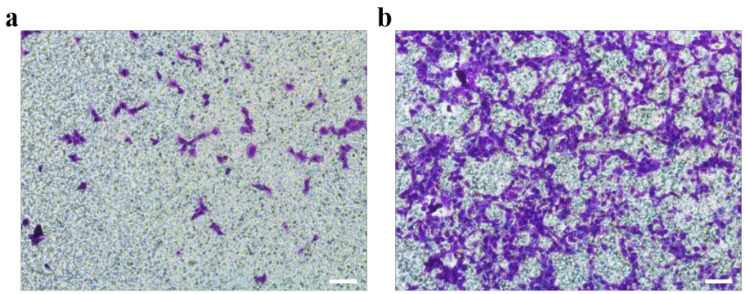
Comparison of metastasis ability between cells cultured in TCP (**a**) and gelatin@NWF (**b**). DU 145 cells were cultured in gelatin@NWF for 7 days. Then cells were collected and tested by Boden chamber assay, scale bar = 50 μm.

Metastasis is one of the characteristics of malignant tumors, which increases the difficulty of tumor cure [44]. Cells grown on gelatin@NWF scaffold for 7 days were harvested to evaluate the cell migration capability by Boyden chamber assay. Compared to 2D cultured cells, more cells were observed on the porous transwell membrane from the cells harvested from the gelatin@NWF scaffold, suggesting a more robust migrating capability (Figure 7).

In summary, from the cell morphology, we observed that cells prefer to form aggregates and spheres on gelatin@NWF scaffold with tailored honeycomb-like pores/wells. The cell growth behavior changes are associated with increased expression of CSC-related marker OCT4, SOX2, and ALDH1A1 at gene and protein levels. The molecular profile changes also lead to a resistance to chemotherapy drugs and an increase of migration capability. The biological characters of the cells grown on the gelatin@NWF scaffold suggest it can be a platform to generate tumor spheres for drug screening and tumor biology research. At the same time, the free-standing and ready-to-use properties of the gelatin@NWF scaffold highlight its potential in those applications.

## 4. Conclusions

A gelatin@NWF hybrid scaffold with tailored micropores structures was prepared by a lyophilizing method. The pre-freezing condition was optimized to modulate the micropore structures on the scaffold. It was found that casting 9% gelatin on NWF scaffold and lyophilizing in liquid nitrogen formed honeycomb-like pores with a size of 10 µm on the gelatin filling. The NWF within the gelatin@NWF hybrid scaffold ensured the mechanical stability for long-term culturing. Through swelling index measurement, we found that the hybrid scaffold promptly took in solution to support cell growth. The cell adhesion and spreading behavior was significantly affected by the size of the micropores on the gelatin@NWF scaffold. We found that tumor cell DU 145 spread on the scaffold with a pore larger than 20 µm. In contrast, cell aggregates and spheroids were observed from a scaffold with a pore size of around 10 µm. Through qPCR examination and Western blot assay, we found that DU 145 spheroids formed on gelatin@NWF prepared in liquid nitrogen had a higher level of SOX2, OCT 4, and ALDH1A1 at mRNA and protein levels. Together, increased levels of those cancer stem cell-related gene expressions lead to stronger resistance to the chemotherapy drug DOX. The cells isolated from the spheroids grown on gelatin@NWF scaffold also show increased motility in Boden chamber migration assay. Moreover, tumor spheroids can be formed on a long-term storage scaffold, highlighting the potential of gelatin@NWF as a ready-to-use scaffold for tumor cell sphere generation and culturing.

## Data Availability

The data in the current study are available from the corresponding authors upon reasonable request.

## Supplementary Materials

The following supporting information can be downloaded at: https://www.mdpi.com/article/10.3390/nano12050808/s1, Figure S1: Digital-image-characterization of lyophilizing gelatin@non-woven fabric; Figure S2: Microscope images of crystal violet stained DU 145 cells grown on gelatin@NWF; Table S1: tensile strength and elongation of films at the break; Table S2: primers and conditions of qPCR.
