# Supplementary material for: Lyophilized Gelatin@non-Woven Scaffold to Promote Spheroids Formation and Enrich Cancer Stem Cell Incidence
Source: Nanomaterials (Basel). 2022 Feb 28;12(5):808. doi: 10.3390/nano12050808 (PMC8912757; doi:10.3390/nano12050808)
Supplement: Supplementary file 1 [file nanomaterials-12-00808-s001.zip › nanomaterials-1549604-supplementary.pdf]

Supplementary information

Lyophilized gelatin@non-woven scaffold to promote spheroids formation and enrich cancer stem cell incidence

Jing-jing Fu [1,2], Feng Chen [2], Hui-hui Chai [2], Li-xia Gao [3], Xiao-hui Lv [2] and Ling Yu [2,4,*]

1 School of Preclinical Medicine, North Sichuan Medical College, Nanchong 637000, China; fujingjing1991@nsmc.edu.cn
2 Institute for Clean Energy & Advanced Materials, School of Materials & Energy, Southwest University, Chongqing 400715, China; cf19950629@email.swu.edu.cn (F.C.); chh0221@email.swu.edu.cn (H.C.); lvxiaohui@email.swu.edu.cn (X.L.)
3 National & Local Joint Engineering Research Center of Targeted and Innovative Therapeutics, Chongqing Key Laboratory of Kinase Modulators as Innovative Medicine, College of Pharmacy & International Academy of Targeted Therapeutics and Innovation, Chongqing University of Arts and Sciences, Chongqing 402160, China; LixiaGao@cqwu.edu.cn
4 Guangan Changming Research Institute for Advanced Industrial Technology, Guangan 638500, China;
* Correspondence: lingyu12@swu.edu.cn

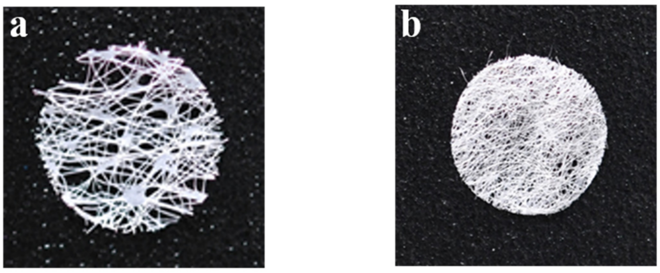

Figure S1. Digital-image-characterization of lyophilizing gelatin@non-woven fabric (NWF) scaffold. (**a**) primary NWF, (**b**) gelatin@NWF.

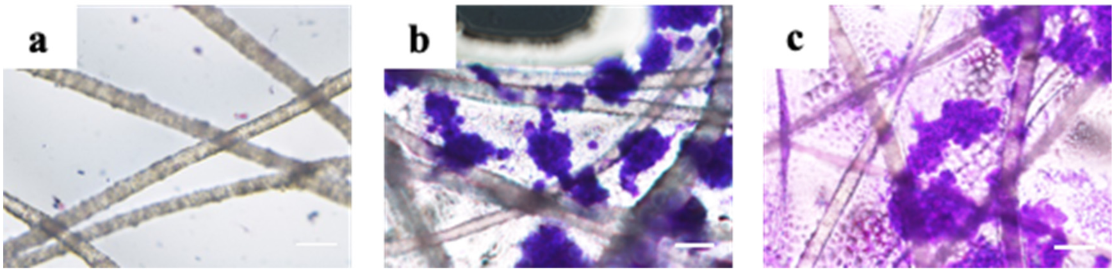

Figure S2. Cristal violet stained DU 145 cells which were grown on (**a**) NWF, (**b**) 6% gelatin@NWF, and (**c**) 12% gelatin@NWF, scale bar=50 μm.

Table S1. Mechanical property of gelatin@NWF

Material	TS (MPa)	EBA%
gelatin film	0.81±0.14	32.12±4.51
gelatin@NWF	0.98±0.25	46.77±2.14

TS: tensile strength; EBA: elongation at break

Table S2. Primers and conditions of qPCR

Gene	Primer	Sequence (5′- 3′)	T_M(℃)
OCT4	forward	AACCGAGTGAGAGGCAACCT	60.0
	reverse	ACAGAACCACACTCGGACCA	60.0
SOX2	forward	TGACCAGCTCGCAGACCTAC	60.0
	reverse	TCGGACTTGACCACCGAAC	60.0
ALDH1A1	forward	CACAGGATCAACAGAGGTTGG	60.0
	reverse	GTCCAAGTCGGCATCAGCTA	56.0
GAPDH	forward	CCAGGTGGTCTCCTCTGACTTCAACA	62.5
	reverse	AGGGTCTCTCTCTTCCTCTTGTGCTC	62.0
